# The Relation between the Fear-Avoidance Model and Constructs from the Social Cognitive Theory in Acute WAD

**DOI:** 10.1155/2016/8281926

**Published:** 2016-11-23

**Authors:** Maria Sandborgh, Ann-Christin Johansson, Anne Söderlund

**Affiliations:** Department of Physiotherapy, School of Health, Care and Social Welfare, Mälardalen University, Box 883, 721 23 Västerås, Sweden

## Abstract

In the fear-avoidance (FA) model social cognitive constructs could add to explaining the disabling process in whiplash associated disorder (WAD). The aim was to exemplify the possible input from Social Cognitive Theory on the FA model. Specifically the role of functional self-efficacy and perceived responses from a spouse/intimate partner was studied. A cross-sectional and correlational design was used. Data from 64 patients with acute WAD were used. Measures were pain intensity measured with a numerical rating scale, the Pain Disability Index, support, punishing responses, solicitous responses, and distracting responses subscales from the Multidimensional Pain Inventory, the Catastrophizing subscale from the Coping Strategies Questionnaire, the Tampa Scale of Kinesiophobia, and the Self-Efficacy Scale. Bivariate correlational, simple linear regression, and multiple regression analyses were used. In the statistical prediction models high pain intensity indicated high punishing responses, which indicated high catastrophizing. High catastrophizing indicated high fear of movement, which indicated low self-efficacy. Low self-efficacy indicated high disability, which indicated high pain intensity. All independent variables together explained 66.4% of the variance in pain disability, *p* < 0.001. Results suggest a possible link between one aspect of the social environment, perceived punishing responses from a spouse/intimate partner, pain intensity, and catastrophizing. Further, results support a mediating role of self-efficacy between fear of movement and disability in WAD.

## 1. Introduction

The fear-avoidance (FA) model [1] describes the disabling process in musculoskeletal pain. It was initially developed to explain the fear dependent development of disability in chronic low back pain [1, 2] but has been generalized to patients with acute [3, 4] and chronic [5] whiplash associated disorder (WAD). In a study on patients with WAD at 4 weeks, three and six months after injury fear-avoidance mediated approximately 20% to 40% of the relationship between pain and disability [3]. The importance of social learning for the development of fear of movement has been highlighted [6] and inclusion of motivational factors in the FA model proposed by several authors [7, 8]. So far, the predictive role of motivational factors in the model has been poorly studied.

Social cognitive theory (SCT) emphasizes the dynamic interaction between the individual, the environment, and behavior [9]. Among several, two motivational factors deriving from SCT could be linked to the FA model: self-efficacy and the input from the interpersonal social environment [6, 7]. Self-efficacy regards the individual's belief in his/her capability to perform a specific behavior needed to achieve a desired outcome [9]. In both acute and chronic WAD functional self-efficacy has a higher impact on disability than fear of movement [5, 10]. Also, studies on primary care patients with persistent pain indicate a connection between catastrophizing, fear-avoidance, specified as fear of movement/(re)injury, and functional self-efficacy [11, 12]. These factors together explain the degree of pain related disability for patients with subacute and chronic pain [11, 13, 14]. Woby and coworkers [15] proposed a revised FA model incorporating self-efficacy as a mediator between fear-avoidance of movement and pain intensity and disability, respectively, in a sample of patients with chronic low back pain. For patients with acute WAD, self-efficacy was found to be a mediator between pain intensity and pain related disability [16]. How self-efficacy relates to the fear-avoidance model when applied to patients with acute WAD remains unclear.

The interpersonal social environment is multidimensional. Of high importance are the interactions with those that an individual frequently encounters in everyday life. Thus, the responses by a significant other, for example, a spouse/intimate partner, to pain communication would influence an individual in pain. There exists, for example, a reciprocity of affect in partners [17], and partner responses to pain expressions are related to psychological distress for individuals with chronic pain [18]. In chronic pain operant conditioning through partner responses could explain both beneficial and nonbeneficial social support [19, 20]. How responses from a spouse/intimate partner are perceived by an individual in pain could therefore influence pain related beliefs and behaviors [6]. Perceived responses from a spouse/intimate partner could activate pathways between potential cues and pain, thus possibly increasing catastrophizing thoughts about movement [8]. In a WAD study [21] a third of the sample perceived low levels of support and solicitous responses from a spouse/intimate partner. These patients also scored low on functional self-efficacy and high on catastrophizing and disability, worsening over time.

With these two factors, self-efficacy and perceived responses from a spouse/intimate partner, we decided to exemplify the possible input from SCT on the FA model. The intention was not to create a new model, but to examine possible additions to the existing FA-model, building on the proposed model by Woby and coworkers [15].

It was assumed that the perceived responses from a spouse/intimate partner would influence patients' interpretations of pain cues, that is, catastrophic thinking, and thus fear-avoidance. Further, that fear-avoidance would be associated with functional self-efficacy, which would in turn be associated with the disability level.

## 2. Methods

### 2.1. Design

A cross-sectional design was used with data from a previous study on patients with acute WAD [16].

### 2.2. Subjects and Procedure

The subjects were recruited at an emergency department at a university hospital. The inclusion criteria were age 18–65, pain in the neck after an accident and ability to understand and write Swedish. Exclusion criteria were a previous neck injury or other chronic pain problems, being unconscious after the accident, ligament injury with instability in the neck, or a vertebral fracture.

Consecutive patients with whiplash-related injury fulfilling the inclusion criteria were asked to participate in the study. A physiotherapist working at the emergency department enrolled the patients into the study, after informed consent, during approximately a one-year period. The enrollment was conducted either before the patients left the emergency department or by phone on the next day. Ninety-five of 132 eligible patients with whiplash injury accepted participating. Patients were sent the questionnaires by mail to be completed at home during the first week after the inclusion and to be returned in prepaid envelopes. For those who failed to answer within two weeks one reminder was done by phone. Sixty-four patients completed the questionnaires. The mean age was 36 years (SD 12.9), 39 women and 25 men. Before the accident 27 patients rated their general health as excellent, 26 very good, 10 rather good, and one bad. According to the Quebec Task Force WAD-classification [22] three patients had WAD grade 0 (no complaint, no physical signs), 14 had WAD grade 1 (neck pain, stiffness, and no physical signs), 43 had WAD grade 2 (neck complaint, musculoskeletal signs), and 4 patients had WAD grade 3 (neck complaint, musculoskeletal signs, and neurological signs).

At the time for data collection an ethical approval was not required for descriptive, nonintervention studies, due to Swedish regulations. All participants signed an informed consent. The study was conducted according to the ethical standards of the Helsinki Declaration for pain research in humans.

### 2.3. Measures

Demographic data were collected with a study specific questionnaire.

Pain intensity (PI) was measured with one rating using an eleven-graded Numerical Rating Scale (NRS), where 0 implied no pain and 10 maximum pain. NRS is considered to be a valid and reliable measure for pain intensity [23].

Perceived responses from a spouse/intimate partner were measured with the support, punishing responses, solicitous responses, and distracting responses subscales from the Multidimensional Pain Inventory-Swedish version (MPI-S) [24–26]. The MPI is a 32-item inventory with in total eight subscales that describe psychosocial, cognitive, and behavioral effects of chronic pain. The subscales' total score varies between 0 and 6. The internal consistency, Cronbach's alpha, was good in the current study for the subscales: support (alpha 0.91), punishing responses (alpha 0.91), solicitous responses (alpha 0.80), and distracting responses (alpha 0.77). The construct validity of MPI-S is good [25].

Catastrophizing thoughts were measured with the catastrophizing (CAT) subscale of the coping strategies questionnaire (CSQ) [27, 28]. The rating is between 0 and 6 per item, and the maximum score is 36. Higher scores indicate a higher degree of catastrophizing thoughts. The Swedish version of the whole CSQ was administered but in this study only the CAT subscale was used. The CAT subscale has shown good reliability in patients with subacute, chronic, and recurring musculoskeletal pain [11].

Fear of movement and (re)injury, mirroring the fear-avoidance construct in musculoskeletal conditions [11, 14], was measured with the Tampa Scale of Kinesiophobia (TSK) [29]. The TSK has 17 items and is rated with a 4-grade format between 1 = “strongly disagree” and 4 = “strongly agree.” The total score range is 17 to 68 with higher scores indicating more fear [11]. The total scores from TSK were used. The Swedish version has good reliability for patients with WAD [30].

Self-efficacy in performing common everyday life activities, that is, functional self-efficacy, was measured with the Self-Efficacy Scale (SES) [11, 31–33]. Participants rated their confidence in performing 20 daily activities in spite of pain. The item rating is 0–10 in which 0 = “not at all confident” and 10 = “very confident.” A total score ranges from 0 to 200. The Swedish version has shown good reliability for patients with WAD [30].

Pain related disability was measured with the Pain Disability Index (PDI) [34–36]. The PDI has seven items, which assess the degree of disability in daily activities. The rating is between 0 and 10 (0 is no interference; 10 is total interference) for each item. A total score ranges from 0 to 70. The PDI is a highly reliable and valid measure for patients with chronic pain [34, 36] and acute WAD [33]. Internal consistency was high, Cronbach's alpha  0.86 in [33]. A Swedish version of PDI was used in the present study [11].

### 2.4. Data Management and Statistical Analyses

All analyses were performed with the Statistical Packages for the Social Sciences, SPSS version 19 for Mac OS X.

Bootstrapping method, simple form, with 1000 samplings was used in all inferential analyses, except for multiple regression analyses, to overcome the assumptions for parametric analyses [37].

Zero-order bivariate correlations were calculated with Pearson's correlation coefficient. One-tailed significance tests were used for bivariate analyses in order to allow MPI subscales a liberal possibility to be included in the model. Missing cases were excluded listwise in all analyses.

A series of simple linear regression analyses [38] were conducted in the order of the FA model and the proposed input in the model from functional self-efficacy and perceived responses from a spouse/intimate partner, in the following order. 

*Analysis 1*. All MPI subscales (support, punishing responses, solicitous responses, and distracting responses) possibly showing significance in bivariate analyses were regarded as dependent variables. Only the punishing responses subscale of MPI-S (dependent variable (DV)) had a significant correlation with pain intensity and was therefore regressed on pain intensity (independent variable (IV)). 

*Analysis 2*. Catastrophizing (dependent variable (DV)) was regressed on punishing responses (independent variable (IV)). 

*Analysis 3*. Fear of movement and (re)injury (dependent variable (DV)) was regressed on catastrophizing (independent variable (IV)). 

*Analysis 4*. Self-efficacy (dependent variable (DV)) was regressed on fear of movement and (re)injury (independent variable (IV)). 

*Analysis 5*. Pain disability (dependent variable (DV)) was regressed on self-efficacy (independent variable (IV)). 

*Analysis 6*. Pain intensity (dependent variable (DV)) was regressed on pain disability (independent variable (IV)). 

The standardized coefficients (beta) are reported for each path in the model as well as significance levels for beta.

Multiple regression analyses were performed to analyze the impact on pain related disability of the presumed independent variables, which had significantly correlated in bivariate analyses. Pain related disability was regressed on fear-avoidance, functional self-efficacy, catastrophizing, and pain intensity as independent variables in the first regression model. In the second model punishing responses were added to the model as an independent variable. In the bivariate analyses this was the only subscale of the MPI subscales in question (support, punishing responses, solicitous responses, and distracting responses) that significantly correlated with pain intensity.

## 3. Results

The means and standard deviations for the study variables are reported in Table 1.

In the bivariate correlation analyses (one-tailed tests) there was a significant correlation between pain intensity and the MPI subscale punishing responses (*r* = 0.37, *p* = 0.02, *n* = 29). The other MPI subscales were not significantly correlated with pain intensity (support *r* = −0.09, *p* = ns, *n* = 39, solicitous responses *r* = −0.19, *p* = ns, *n* = 25, and distracting responses *r* = −0.17, *p* = ns, *n* = 25). Punishing responses subscale was significantly correlated with catastrophizing (*r* = 0.47, *p* = 0.005, *n* = 30). A chain of relations emerged; catastrophizing was significantly correlated with fear of movement and (re)injury (*r* = 0.52, *p* < 0.001, *n* = 48). Fear of movement and (re)injury was significantly correlated with functional self-efficacy (*r* = −0.42, *p* = 0.002, *n* = 45). Functional self-efficacy was significantly correlated with pain disability (*r* = −0.73, *p* < 0.001, *n* = 45), and finally pain disability was significantly correlated with pain intensity (*r* = 0.56, *p* < 0.001, *n* = 45).

A series of simple linear regression analyses were conducted in an order based partly on the original fear-avoidance model and partly regarding the possible role of functional self-efficacy and perceived partner responses (punishing responses) in this model.

*Analysis 1*. Pain intensity explained 11% of the variance in punishing responses (Adj *R* ^2^ = 0.11, *p* = 0.046). 

*Analysis 2*. Punishing responses explained 19% of the variance in catastrophizing as dependent variable (Adj *R* ^2^ = 0.19, *p* = 0.009). 

*Analysis 3*. Catastrophizing explained 25% of the variance in fear of movement and (re)injury (Adj *R* ^2^ = 0.25, *p* < 0.001). 

*Analysis 4*. Fear of movement and (re)injury explained 16% of the variance in functional self-efficacy (Adj *R* ^2^ = 0.16, *p* = 0.004). 

*Analysis 5.* Functional self-efficacy explained 52% of the variance in pain disability (Adj *R* ^2^ = 0.52, *p* < 0.001). 

*Analysis 6*. Pain disability explained 30% of the variance in pain intensity (Adj *R* ^2^ = 0.30, *p* < 0.001). 

**Figure 1:**
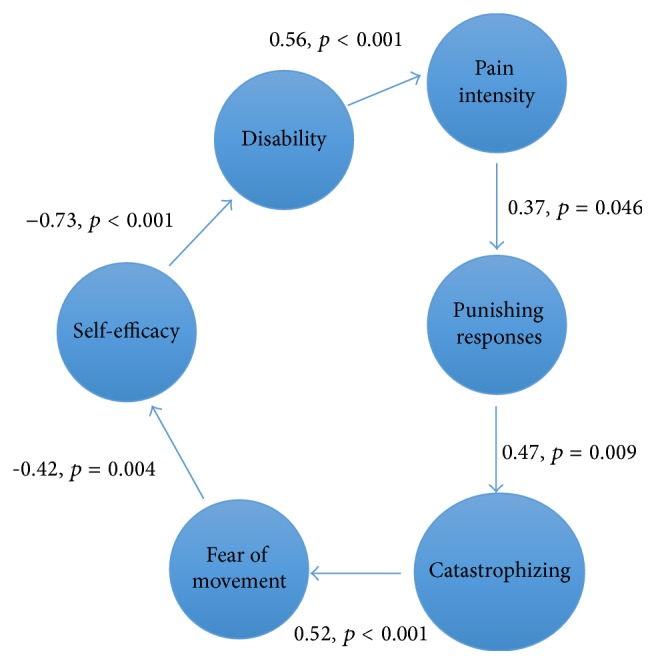
Results in beta and *p* values from linear regression analyses based on the modified fear-avoidance model including functional self-efficacy and the interpersonal environment; punishing responses from a spouse/ intimate partner.

Figure 1 describes the proposed input of functional self-efficacy and perceived partner responses in the fear-avoidance model, including beta and *p* values for each path.

The results of analyses with or without bootstrapping were identical; that is, coefficients' significance level did not change by bootstrapping. Thus, the reported results are from nonbootstrap analyses.

The first multiple regression model included the predictors pain intensity, catastrophizing, fear of movement and (re)injury, and self-efficacy, and a significant model emerged, *p* < 0.001, that explained 63.9% of the variance in pain disability. The second model included the predictors pain intensity, catastrophizing, fear of movement and (re)injury, self-efficacy, and punishing responses from a spouse/intimate partner. This model explained 66.4% of the variance in pain disability, *p* < 0.001.

## 4. Discussion

We used available data from a previous study on patients with acute WAD [16] to explore a possible connection between the FA model and SCT. This was an attempt to exemplify how constructs from SCT could be integrated with the FA model. By simple linear regressions a possible pathway was demonstrated, from pain intensity to perceived (punishing) responses from a spouse/intimate partner, catastrophizing, fear of movement, functional self-efficacy, and disability for patients with acute WAD. The results of multiple regression analyses showed that, in a FA model which included functional self-efficacy, perceived punishing responses could add to the model in explaining pain related disability. This could imply that if a person in pain perceives responses from a spouse/intimate partner to be somehow punishing this can contribute to increased catastrophizing. In turn, catastrophizing can increase fear of movement, leading to a decreased functional self-efficacy, resulting in higher disability and probably increased pain intensity. The results may broaden the perspective in relation to the FA model by reflecting the complex interplay between several contributing factors such as self-efficacy beliefs and how responses from others in the closest social environment are interpreted. This probably mirrors the multifactorial and interactive process when people in pain develop disabling fear of movement.

The order of the steps for the analyses was based on earlier research and theoretical considerations in contrast to a statistical solution. As our aim was to exemplify an expansion of the existing model the order was according to the steps in the FA model, with the proposed input from additional variables. The beta coefficients in the model (Figure 1) were either medium or large and significant, which can be interpreted as strengthening the model's validity. The shared variance between variables in the regression analyses varied between 11% and 52%. Obviously, there are both weaker and stronger relationships in our model. Pain intensity, punishing responses, and catastrophizing as well as fear of movement and functional self-efficacy had the lowest shared variances. However, earlier studies have shown that catastrophizing, fear-avoidance, and self-efficacy have an important role in the original fear-avoidance model [1, 5, 16, 39]. Thus, we believe that our results point to the same direction as earlier studies, despite low shared variances. Further, the low shared variance implies that the measured variables are conceptually distinct.

To our knowledge, in no other studies the links between perceived responses from a spouse/intimate partner and the FA model have been investigated. Of the possible types of responses we included, only the predictive value of perceived punishing responses could be confirmed. In the model it appears that pain intensity predicts perceived punishing responses, which in turn predict catastrophizing thoughts. In a study of chronic WAD [5] catastrophizing played a significant role in predicting disability. It is possible that higher pain intensity is to a higher degree demonstrated in pain behaviors and expressions and thus could cause negative responses from a partner [6, 17]. This would then affect the patient's thoughts on what pain means, that is, catastrophizing thoughts. Perceived punishing responses from a spouse/intimate partner, for example, a partner getting angry and irritated, have been shown to be associated with a higher degree of pain related disability [21]. Solicitous responses from a spouse/intimate partner could have an influence with similarities to punishing responses and further drive fear and avoidance [20]. However, the impact of solicitous responses on fear-avoidance could not be demonstrated in our study.

The results confirmed the importance of social cognitive factors in WAD and are regarding self-efficacy in line with studies on acute [10] and chronic WAD [5]. Woby et al. [12] suggested a mediational role of self-efficacy between fear-avoidance and pain related disability. The TSK is a composite measure of both fear and avoidance of movement and was used as an entity in our model. We could see an impact of fear of movement on self-efficacy but the supposed mediational role of self-efficacy between fear and avoidance could not be studied. The exact relation between fear of movement and self-efficacy needs further study.

This study implies the necessity of assessing not only pain intensity and disability, but also perceived punishing responses from a spouse/intimate partner, catastrophizing, fear of movement, and functional self-efficacy in patients with acute WAD. Individuals rating high in perceived punishing responses from a spouse/intimate partner, catastrophizing, and fear of movement and low in functional self-efficacy should get more attention from health care since their pain problems might result in increased disability and chronicity. In WAD disability and pronounced fear-avoidance is predictive for the translation from the acute to the chronic phase [40]. According to Söderlund and Lindberg [41] catastrophizing was the strongest predictor of increased disability in WAD six weeks to one year after accident and was a mediator between functional self-efficacy and disability [42]. Nederhand et al. [40] showed that high initial fear of movement in patients with acute WAD predicted high disability at six-month follow-up. In a study on patients with WAD those classified as “dysfunctional” rated the highest scores in the MPI subscale punishing responses at inclusion three months after the accident. These patients also had the highest mean in disability at the one-year follow-up [21]. Further, low functional self-efficacy in the acute phase was shown predictive for disability at the six-month follow-up after the accident [33], and in a cross-sectional study low functional self-efficacy was related to disability [5]. Our study is the first in combining earlier results and incorporating interpersonal environmental variables in the fear-avoidance model, in accordance with the SCT.

We used fairly simple analyses, which could be a limitation in this study. However, in mediation analyses linear regressions are recommended as a first step, prior to structural equation modeling that claims larger samples [38]. For further studies mediator analyses should be used, preferably in large datasets, to confirm the order of added variables in the FA model. This was a cross-sectional study and longitudinal studies are needed to establish not just a statistical predictive value but also causal relationships in the modified FA model for the acute to chronic process in WAD.

The small sample size can be seen as a limitation. However, demographic data were similar to earlier studies with reference to age [5, 32, 33], gender [5, 21, 32], WAD grade [5, 33], level of pain intensity [32, 33], disability [33], functional self-efficacy [32, 33], fear of movement, and catastrophizing [33], suggesting comparability with previous studies regarding sample constitution.

We used MPI subscales as a measure of perceived responses of a spouse/intimate partner, in MPI called a “significant other.” During the time for data collection the MPI still defined a significant other as a family member such as a spouse. As a consequence not all participants answered the MPI due to not having a significant other as defined. In future studies the newer version of MPI, with a broader definition of significant other, should be used, hopefully leading to less missing data [43].

The MPI only asks about how a person in pain perceives these responses and does not include the more dynamic interaction between, for example, partners. To mirror the interaction between the individual and a spouse/intimate partner and how this affects behavior, more data is needed: how a spouse/intimate partner report their responses [44], observations of interactions, and the consequences of different types of responses [17].

Our results indicate the importance of perceived punishing responses from a spouse/intimate partner and patients' functional self-efficacy, and in consequence these factors might also be valuable to consider in the treatment of WAD patients. In sum, in spite of its limitations, the present study provides some support for integrating concepts from the social cognitive theory with the fear-avoidance model. The functional self-efficacy and a limited aspect on the interpersonal social environmental, that is, perceived punishing responses from a spouse/intimate partner, can contribute with important information to the fear-avoidance model in patients with acute WAD. Thus, combining concepts from social cognitive theory and the fear-avoidance model may be fruitful to increase our understanding of the complex pain related disability process in WAD. Further studies are however needed to deny or confirm our results.

## Figures and Tables

**Table 1:** Means and standard deviations for the studied variables in the sample.

Measure	Mean (SD)
*Pain intensity* (NRS) (0–10)	4.8 (2.9)
*n* = 61	
*Multidimensional pain inventory* (MPI-S) (0–6)	
Punishing responses, *n* = 32	0.6 (1.0)
Distracting responses *n* = 26	3.3 (1.7)
Support, *n* = 43	4.9 (1.3)
Solicitous responses, *n* = 26	2.9 (1.5)
*Catastrophizing* (CAT) (0–36)	6.3 (5.6)
*n* = 48	
*Fear of movement and (re)injury* (TSK) (17–68)	33.3 (9.9)
*n* = 53	
*Self-efficacy scale *(SES) (0–200)	135.7 (43.7)
*n* = 45	
*Pain disability index* (PDI) (0–70)	24.4 (18.0)
*n* = 49	
